# The Implications of Nutritional Strategies that Modify Dietary Energy and Lysine for Growth Performance in Two Different Swine Production Systems

**DOI:** 10.3390/ani10091638

**Published:** 2020-09-11

**Authors:** Pau Aymerich, Carme Soldevila, Jordi Bonet, Josep Gasa, Jaume Coma, David Solà-Oriol

**Affiliations:** 1 Vall Companys Group, 25191 Lleida, Spain; csoldevila@vallcompanys.es (C.S.); jbonet@vallcompanys.es (J.B.); jcoma@vallcompanys.es (J.C.); 2 Animal Nutrition and Welfare Service, Department of Animal and Food Sciences, Universitat Autònoma de Barcelona, 08193 Bellaterra, Spain; Josep.Gasa@uab.cat (J.G.); David.Sola@uab.cat (D.S.-O.)

**Keywords:** lysine, energy, growing pig, finishing pig, castration

## Abstract

**Simple Summary:**

Reducing dietary energy is a common practice for dealing with the price volatility of high energy sources, such as fats and oils, which are the costliest constraints in swine feed formulation. Theoretically, pigs can overcome a reduced energy density by increasing feed intake; however, as other factors like fibrous ingredients limit feed intake physically rather than metabolically, reducing dietary energy could also entail a lower energy intake. The expected effect on feed intake also influences lysine intake, and therefore, when NE trials are conducted, it is necessary to ensure that lysine is not a limiting factor for growth. In the present work, the effects of two dietary energy and lysine levels were tested in a factorial arrangement. The same approach of different levels was analyzed in two different swine production systems targeting different carcass traits. The experiment showed that in one system, reducing energy density did not impair growth; however, in the other system, it limited growth slightly by limiting fat deposition. Although reducing energy density increased feed intake, pigs could not reach a similar energy intake, and consequently were more efficient using energy for growth.

**Abstract:**

This work aimed to determine the impacts of lowering dietary net energy (NE) density in two swine production systems that produce pigs with different carcass traits. To ensure that dietary lysine was not limiting growth, two studies were conducted in a 2 × 2 factorial arrangement with NE and standardized ileal digestible lysine (SID Lys) as experimental factors. A total of 1248 pigs were used in each study, Pietrain (Exp. 1, males non-castrated) or Duroc (Exp. 2, males castrated) sired. Reducing NE resulted in a greater feed intake; however, this was not sufficient to reach the same NE intake. While in Exp. 1 a 3.2% lower NE intake did not impair average daily gain (ADG; *p* = 0.220), in Exp. 2 a 4.7% lower NE intake reduced ADG by 1.4% (*p* = 0.027). Furthermore, this effect on ADG entailed a reduced ham fat thickness (*p* = 0.004) of the first marketed pigs. Increasing SID Lys only had a positive effect in Exp. 1, but no significant interaction between NE and SID Lys was reported (*p* ≥ 0.100). Therefore, dietary NE can be reduced without impairing growth performance when pigs can increase feed intake sufficiently, and thus, limit energy deficiencies.

## 1. Introduction

It is necessary to have a thorough understanding of the consequences of modifying dietary energy and lysine in each specific pig production context because energy and amino acids are the most expensive constraints in feed formulation. In Europe, the swine industry involves two main production systems depending on the sire line used and the entire/castrated status of males. These systems result in carcasses with different fat/lean depositions and qualities in relation to the requirements of the packing and curing industries [1]. For the market that requires high lean carcasses, highly conformed sire lines such as Pietrain [2,3] without male castration are preferred. In contrast, for the market that requires a minimum fat deposition [4], high feed intake sire lines (Duroc or synthetic lines) are used, with males usually castrated [5,6]. Thus, to maximize performance and pork quality while meeting the processors’ requirements, each sire line and sex combination [7,8,9] needs tailored nutritional programs [6,10].

Adjusting dietary energy density is a common practice for dealing with the price volatility of high energy ingredients (fats and oils) [11]. When it is necessary to minimize the cost of feed, it is possible to reduce energy density without negatively impacting average daily gain (ADG), because pigs increase their average daily feed intake (ADFI) to reach a similar energy intake [12,13]. However, in some conditions, pigs cannot completely compensate for the reduced energy density, especially younger animals [14,15,16] or low-weight pigs [17] with a limited ADFI [18]. Under these circumstances, the lower energy intake may impair ADG by limiting protein [19,20] or fat deposition [21,22,23], depending on whether pigs are in the energy or amino acid-dependent phase [24]. In the systems for producing lean carcasses, the effects of reducing dietary energy density could be problematic when protein deposition is limited; however, in systems requiring a minimum fat deposition, the effect may be a problem when fat deposition is reduced. In a meta-regression analysis, Nitikanchana et al. [25] showed that increasing dietary energy density resulted in greater ADG if dietary lysine was not limiting. Therefore, it is relevant to work at the amino acids levels which do not limit growth performance if we want to evaluate the energy effects. Marçal et al. [26] suggested that energy trials should formulate diets based on the standardized ileal digestible lysine to net energy ratio (SID Lys:NE) instead of SID Lys to report effects on ADG. However, when pigs cannot completely compensate for a reduced energy density [14], then the lower energy intake will entail a reduced SID Lys intake [27] that may limit growth performance [28].

The hypothesis of the present work was that reducing net energy (NE) will not impact growth performance when SID Lys intake is not limiting if pigs are able to match the same NE intake in the two abovementioned systems. Therefore, the aim of this work was to determine the effects of reducing dietary net energy (NE) and their interaction with dietary SID Lys on growth performance in two different swine production systems targeting specific products.

## 2. Materials and Methods

### 2.1. Experiment 1: Pietrain

The aim of this experiment was to determine whether reducing NE had an impact on growth performance of lean pigs (20–40 kg) and evaluate if dietary SID Lys modified the possible effects. A further aim was to evaluate the carryover effects when lean pigs are fed a common diet (40–110 kg).

#### 2.1.1. Experimental Design and Animals

In this study, the effects of dietary NE and SID Lys on growth performance of lean growing pigs was analyzed in a 2 × 2 factorial arrangement. The treatments represented the factorial combination of one of two SID Lys levels (1.00 vs. 1.20%) and NE levels (2360 vs. 2550 kcal NE/kg). At arrival, a total of 1248 pigs (Pietrain × (Landrace × Large white), half boars and half gilts) were grouped by body weight (BW) in 96 non-mixed sex pens of 13 pigs. After a 9 day adaptation period, pigs were individually weighed (19.7 ± 3.8 and 20.0 ± 4.1 kg, mean ± SD, boars and gilts, respectively) and each pen was allocated to one of the four dietary treatments, with twenty-four replicates per treatment. Pens were classified in one of three BW blocks (large, medium, or small). Each pen (3 × 3 m) had a half slatted concrete floor, 1-hole wet-dry Maxi Grow Feeder (Rotecna, Agramunt, Spain) and an additional nipple waterer on the other side. Ad libitum access to feed and water was ensured during the entire trial. Pigs were individually weighed and monitored using electronic ear tags at the beginning of the trial, and at day 14, 26, 68 and 116, when the experiment finished. Feed intake was measured weekly on a pen basis considering the feed offered and measuring the remaining feed in each feeder. In addition, when a pig was removed due to illness or death, the feed intake was corrected for the days in the week that the pig was not in the pen.

#### 2.1.2. Feeding and Analyses

The pigs were fed a common commercial diet (1.17% SID Lys and 2500 kcal NE/kg) in the nine days prior to starting the experiment. Afterwards, during the first 26 days of the experiment, pigs were fed the four experimental diets, based on maize, wheat and soybean meal (Table 1). To reduce dietary NE, animal fat inclusion was reduced, whereas wheat middlings were increased. In addition, SID Lys was increased by modifying the inclusion of crystalline amino acids. Feed was produced in successive blending batches (5000 kg), and 2% animal fat was added post-pelleting to ensure a good quality pellet. After pelleting, feed samples were collected for each blending batch and analyzed for crude protein (ISO 16634-2:2016) and crude fat (Commission Regulation (EC) No 152/2009 of 27 January 2009) before being used to ensure no relevant deviations from the calculated values. Furthermore, the AA composition (Method 994.12) [29] was posteriorly analyzed in a blend of the different batches of each experimental feed. From day 26 onwards, all pigs were fed the same feeds: one feed (0.95% SID Lys and 2440 kcal NE/kg) from day 26 to 68 and the other feed (0.84% SID Lys and 2450 kcal NE/kg) from day 68 to 116. During the entire trial, feeds were distributed in the different pens using an automatic feeding system (DryExact Pro; Big Dutchman, Vechta, Germany). For the low SID Lys diets, the cost of feed ingredients was 253 and 268 EUR/t for the low and high energy treatments, respectively. For the high SID Lys feed, the cost of feed ingredients was 265 and 280 EUR/t for the low and high energy treatments, respectively. The cost of feed ingredients in the common feed phases was 225 EUR/t for the period 26–68, whereas for the last period (68–116) it was 215 EUR/t.

#### 2.1.3. Calculations and Statistical Analysis

Pen BW, ADG, ADFI, NE daily intake, SID daily intake, feed to gain ratio (F/G), NE efficiency per kg gain (NEE), SID Lys efficiency per kg gain (LysE), and feed cost per kg gain were calculated per pen for the initial phases (phase 1: 20–29 kg BW—d 0 to 14, phase 2: 29–38 kg BW—d 14 to 26). In addition, BW, ADG, ADFI and F/G were calculated for the period when pigs were fed a common diet. The average ADG and ADFI were calculated for phases 1–2, and overall weighting according to the number of days in each subphase. The other variables were then recalculated using the ADG and ADFI. Statistical analysis was carried out with R [30]. Linear mixed models, initially including SID Lys, NE, BW block and sex, and all the possible interactions, were initially fitted using the nlme package [31] considering room as a random effect. The models were assessed using type III ANOVA. Only interactions that were significant (*p* ≤ 0.05) for at least one variable were included in the final model. The emmeans package [32] was used to calculate the least square means.

### 2.2. Experiment 2: Duroc Sire Line

The aim of experiment 2 was to determine the effects of reducing NE in different dietary SID Lys levels on the growth performance of Duroc pigs, which have a high feed intake capacity, and the effects of NE on carcass composition. The experiment was divided in two subphases (30–75 and 75–120 kg BW) in which the main differences were the SID Lys levels tested and the low NE value.

#### 2.2.1. Experimental Design and Animals

We used a 2 × 2 factorial design to analyze the effects of NE and SID Lys. From 30 to 75 kg BW pigs were fed either 0.94 or 1.04% SID Lys and 0.80 or 0.90% SID Lys from 75 to 120 kg BW. Regarding NE, pigs were fed either 2450 kcal NE/kg or a low energy diet that was 2200 and 2230 kcal NE/kg for the first and second subphase, respectively. A total of 1248 pigs (Duroc × (Landrace × Large white), half barrows and half gilts) were randomly grouped in non-mixed sex pens of 13 animals according to their initial body weight. After a 10 day adaptation period, pigs were individually weighed (33.5 ± 5.0 and 31.4 ± 4.9 kg, mean ± SD, barrows and gilts, respectively) and the pens were randomly allocated to one of the four dietary treatments, with twenty-four replicates per treatment, and classified in three BW blocks. Each pen (3 × 3 m) had a half slatted concrete floor, 1-hole wet-dry Maxi Grow Feeder (Rotecna, Agramunt, Spain) and an additional nipple waterer on the other side. Ad libitum access to feed and water was ensured during the entire trial. Pigs were individually weighed and monitored using electronic ear tags at day 0, 23, 43, 72 and 85, when the experiment finished. Feed intake was measured weekly on a pen basis considering the feed offered and measuring the remaining feed in each trough. In addition, when a pig was removed due to illness or death, the feed intake was corrected for the days in the week that the pig was not in the pen. The day after the experiment finished, a total of 380 pigs (half barrows and half gilts) from the large BW block, representing the first marketing group, were moved to the slaughterhouse (Cárnicas Cinco Villas, Ejea de los Caballeros, Spain). Pigs of each sex were divided into two groups, depending on whether they came from the high or low NE treatments. Individual carcasses were weighed, and carcass composition was measured using Autofom III (Frontmatec Food Technology, Kolding, Denmark). The measured parameters included hot carcass weight (HCW), carcass leanness (CL), backfat thickness (BFT) and ham fat thickness (HFT). Carcass leanness (%) was automatically calculated from 9 measurements provided by the 16 ultrasound transducers using the official equations for grading carcasses in Spain (2012/384/UE).

#### 2.2.2. Feeding and Analyses

During the 10 day adaptation period, pigs were fed a common commercial diet (1.08% SID Lys diet and 2475 kcal NE/kg). The experiment was divided into two phases, one from day 0 to 43 (growing phase) and one from day 43 to 85 (finishing phase). In both phases, the pigs remained in the same treatment, that is, one of the combinations of high or low SID Lys and high or low NE. In the growing phase dietary treatments, SID Lys was increased using crystalline AA. To reduce NE, the inclusion of animal fat was reduced, while part of wheat was replaced by wheat middlings and barley. Similarly, in the finishing diets, dietary NE was limited by reducing the amount of palm oil included, while maize was replaced by barley and wheat middlings, and part of the soybean meal by sunflower meal. As in the initial feeds, differences in dietary SID Lys were the result of modifying the inclusion of crystalline AA (Table 2). Feed was produced in successive blending batches (5000 kg), and in the high NE diets, a 2.0–1.5% of the fat source, for growing and finishing feeds, respectively, was applied post-pelleting to ensure a good quality pellet. After pelleting, feed samples were collected for each blending batch, and crude protein (ISO 16634–2:2016) and crude fat (Commission Regulation (EC) No 152/2009 of 27 January 2009) were analyzed before use to ensure that levels were similar to those calculated. Furthermore, AA composition (Method 994.12) [29] was analyzed in a blend of the different batches of each feed. During the entire trial, feeds were distributed in the different pens using an automatic feeding system (DryExact Pro; Big Dutchman, Vechta, Germany).

#### 2.2.3. Calculations and Statistical Analyses

Pen BW, ADG, ADFI, NE daily intake, SID daily intake, F/G, NEE, LysE, and feed cost per kg gain were calculated per pen for four phases (days 0–23, 23–43, 43–72, 72–85). Data from days 0–43 (growing phase) were combined because there was a health challenge that increased the variability in the results after the first 23 days. In addition, data from days 43–85 days were also combined in a single period (finishing phase) because of the short duration of the last period. The average ADG and ADFI were calculated for each phase weighting by the number of days in each subphase. The other variables were then recalculated using the ADG and ADFI. Statistical analyses were carried out with R [30]. Linear mixed models initially including SID Lys, NE, BW block, sex, and all the possible interactions were initially fitted using the nlme package [31] considering room as a random effect. Afterwards, interactions that were never significant (*p* > 0.05) were removed from the final models. Then, type III ANOVA was performed using the same package for each variable. The emmeans package [32] was used to calculate least square means. The effect of NE on carcass traits of first marketed pigs was analyzed using linear models [30]. To predict CL, BFT and HFT, HCW was included as a covariate in the model. The interaction of HCW with NE was initially tested, and if not significant (*p* > 0.050), it was included just as a linear predictor of carcass traits.

## 3. Results

### 3.1. Experiment 1: Pietrain

The analyzed content of the feeds was close to that calculated, and only some small deviations in the CP content were reported for some dietary treatments; however, they were within the error of the analytical method (±0.50% CP). In addition, the amino acid profile did not show any significant deviations in the amino acid composition of the feeds. Furthermore, the final statistical model only included the double interactions between SID Lys, NE, BW block and sex. Triple and quadruple interactions were not included as they were never significant (*p* > 0.05).

#### Effects on Growth Performance

The effects of dietary NE and SID Lys on growth performance are reported in Table 3. There was no evidence of an interaction between SID Lys and NE for any of the analyzed variables (*p* ≥ 0.100). Pigs were 0.7 (*p* = 0.003) and 1.1 kg heavier (*p* < 0.001), on day 14 and 26, respectively, when SID Lys was increased, but no evidence of a NE effect on BW was observed (*p* ≥ 0.331) from day 0 to 26. Pigs fed the high SID Lys diets showed a greater ADG in both initial phases (*p* < 0.001) due to a reduced F/G (*p* < 0.001), as no evidence of differences in ADFI (*p* ≥ 0.821) was reported. Thus, increasing 20% dietary SID Lys resulted in a 20% greater SID Lys intake (*p* < 0.001) but only a 6.4% increase in ADG overall phases 1 and 2. The NEE was also reduced (*p* < 0.001) in a similar proportion to F/G, whereas LysE significantly increased (*p* < 0.001); thus, SID Lys efficiency for growth was worsened. Feed cost per kg gain was significantly reduced in phase 1 (*p* = 0.006), but there was no evidence of a reduction in phase 2 (*p* = 0.266). Reducing 190 kcal NE/kg by removing 3.5% added animal fat did not impact significantly ADG in either of the experimental phases (*p* ≥ 0.220). Although the ADFI of pigs in the low NE diet was on average 0.05 kg greater (*p* < 0.001) than the ADFI of pigs fed the high NE diet, the calculated NE intake was greater in the high NE diet (*p* < 0.001). As a result of the higher ADFI but lower NE intake, F/G improved, whereas NEE was worsened (*p* < 0.001) when NE was increased. Similarly, feed cost per kg gain was lower for the 2360 kcal NE/kg diets for each phase and overall (*p* ≤ 0.004).

When fed a common diet, pigs previously in the high SID Lys treatments remained heavier at day 68 (*p* = 0.023), but the same difference was not significant at the end of the experiment (*p* = 0.103). Interestingly, although initially there was no evidence of a difference in BW in relation to NE (*p* = 0.331), at day 116, pigs that from day 0–28 had been fed the low NE diets were 1.3 kg heavier than pigs in the high NE diet (*p* = 0.025). The increased BW was the result of a greater ADFI (*p* = 0.001) and consequently a greater ADG (*p* = 0.037). A tendency for a poorer F/G in pigs previously fed the low SID Lys diets was also observed (*p* = 0.059). Similarly, a poorer F/G was reported for pigs previously fed the high NE diet (*p* = 0.024). The effects of the initial dietary treatments were also evaluated in relation to the overall growth performance. Similarly, as for BW, increasing SID Lys from day 0 to 26 did not increase ADG significantly (0.787 vs. 0.780 kg; *p* = 0.106). However, a greater ADG was reported for pigs initially fed the low NE diet (0.789 vs. 0.778 kg; *p* = 0.020). Nevertheless, pigs initially fed the high NE diet showed an improved F/G (*p* < 0.001) due to a 0.050 kg lower ADFI (*p* < 0.001), which was a huge difference compared to the effect on ADG. Increasing SID Lys did not impact the overall ADFI (*p* = 0.491) and F/G (*p* = 0.162). However, a tendency for an interaction between SID Lys and NE on F/G (*p* = 0.100) was reported because in the high NE diet increasing SID Lys reduced feed efficiency (2.07 vs. 2.05; *p* = 0.033), whereas in the low NE diet, it had no effect (2.09 vs. 2.09; *p* = 0.857). Finally, the overall results did not show any effect of initially fed SID Lys (*p* = 0.310) or NE (*p* = 0.454) on feed costs per kg gain.

### 3.2. Experiment 2: Duroc

Crude protein, crude fat and amino acid analyzed composition were consistent with the calculated values, and only some deviations were observed for Met + Cys and Thr in the low SID Lys high NE diet in the growing phase. The quadruple interactions were removed from all the final statistical models because those were not significant (*p* ≥ 0.247), and only the triple interaction between SID Lys, BW block and sex was left in the model as it was significant for some variables in the growing phase and overall.

#### 3.2.1. Effects on Growth Performance

Table 4 reports the factorial effects of NE and SID Lys density on growth performance. Increasing SID Lys had no significant effect on BW on any measurement day (*p* ≥ 0.644); however, reducing NE reduced BW by 1.3 kg BW at day 72 (*p* = 0.039) and tended to reduce BW by 1.2 kg at the end of the experiment (*p* = 0.077). No evidence of an interaction between SID Lys and NE was reported for BW (*p* ≥ 0.194). From day 0 to 43, a tendency for a significant interaction between SID Lys and NE was only reported for SID Lys intake (*p* = 0.071) because the effect of increasing SID Lys was greater in the low NE diets than in the high NE diets. However, no interaction was reported for ADG or F/G. Increasing SID Lys had no significant impact on growth performance, and only increased SID Lys intake (*p* < 0.001), SID Lys per kg gain (*p* < 0.001) and feed cost per kg gain (*p* < 0.001). In contrast, reducing NE did impact all analyzed variables (*p* ≤ 0.004) except for ADG (0.963 vs. 0.978 kg; *p* = 0.105). As expected, when NE density was reduced, ADFI, F/G and LysE increased, whereas NE intake, feed cost per kg gain, and NEE decreased. In the finishing phase (days 43–85), no interaction between SID Lys and NE was observed (*p* ≥ 0.141). As reported in the growing phase, SID Lys did not have a positive impact on growth performance, except for a greater SID Lys intake, LysE and feed cost per kg gain (*p* < 0.001). Similarly, reducing NE influenced all variables except ADG (*p* = 0.220). In contrast with the growing phase, reducing NE negatively impacted feed cost per kg gain in the finishing phase (0.608 vs. 0.630 EUR/kg; *p* < 0.001) as the differences in cost were smaller, but the increase in ADFI was greater than in the growing phase. As a result of the greater ADFI of barrows compared to gilts (3.32 vs. 3.00 kg; *p* < 0.001), the effect of increasing SID Lys on SID Lys intake was greater in barrows than in gilts (*p* = 0.023). Overall, there was no evidence that increasing SID Lys improved growth performance. Contrarily to the analysis by phases, reducing NE impaired ADG (1.030 vs. 1.045 kg; *p* = 0.027) with a greater feed cost per kg gain (0.560 vs. 0.553 EUR/kg; *p* = 0.004).

#### 3.2.2. Effects on Carcass Traits

**Figure 1:**
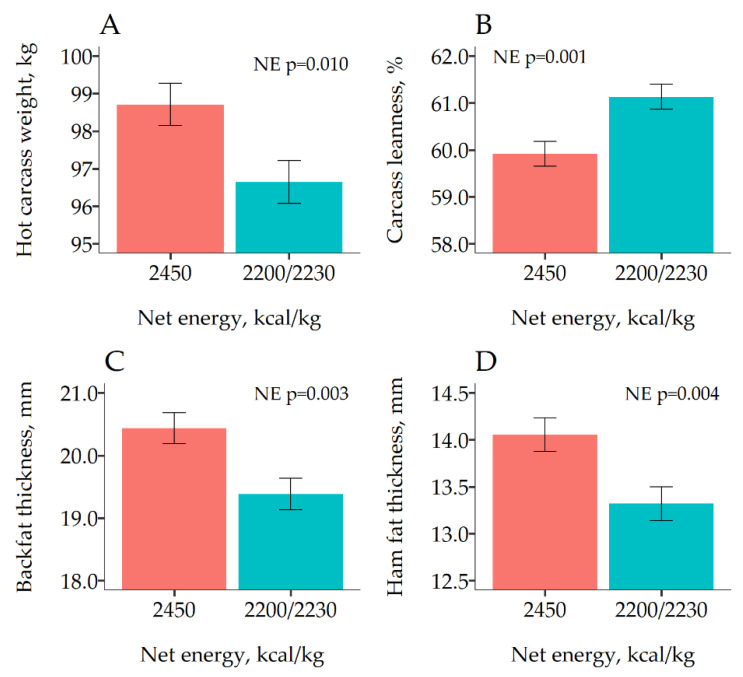
Least square means of the effect of dietary net energy (NE) on carcass traits measured on the 380 first marketed pigs at day 86 of Experiment 2. For B, C and D, the response was adjusted using carcass weight (HCW) as a linear predictor. Error bars represent the standard error of the mean.

The results of the effects of dietary NE on carcass traits of first marketed pigs are shown in Figure 1. As expected, pigs fed the high NE diets were heavier (*p* = 0.005). In addition, reducing the NE content in the diet increased CL (61.1 vs. 59.9%). Increasing HCW reduced CL 0.105%/kg (*p* < 0.001), but there was no evidence that this effect was different between the two NE densities (*p* = 0.314). Reducing dietary NE concentration also reduced significantly BFT (19.4 vs. 20.4 mm; *p* =0.003) and HFT (13.3 vs. 14.1 mm; *p* = 0.004).

## 4. Discussions

Energy and amino acids represent the costliest constraints in feed formulation. It is necessary to determine the impact of reducing energy density on practical swine nutrition to ascertain the extent these constraints can be reduced without negatively impairing growth performance. Grow-finishing pigs can compensate a low energy density diet by increasing ADFI [13]. However, there are inconsistencies regarding whether reducing dietary energy density results in a reduced [14,16,27,33] or similar energy intake [19,22,34]. If the energy value of feeds is correctly valued, and pigs can compensate for the reduced energy density, it would be expected that there would be no effect on ADG if there are no differences in maintenance energy requirements [35]. Moreover, when SID Lys (first limiting AA used as a reference in the ideal protein concept) is formulated on a ratio with energy, pigs will have a similar SID Lys intake [16]. Nevertheless, when a lower energy density impairs energy intake and diets are formulated based on a ratio between SID Lys and energy, then pigs in the low energy diets also have a lower SID Lys intake [26,27]. Therefore, sometimes it is difficult to discern whether the effects of reducing NE on ADG are due to energy or to lysine being limiting.

The SID Lys levels used in the experiments were chosen based on previous trials [18] and on published nutrient requirements [35]. As Pietrain sire line was expected to have a lower ADFI than a Duroc sire line, the differences in dietary SID Lys were greater in Exp. 1 than in Exp. 2 in order to achieve a similar effect on daily SID Lys intake. In Exp. 1 NE densities were chosen based on the available literature, reporting no effect of reducing NE concentration from 2.51 to 2.34 Mcal/kg on ADG or NE intake [34]. In contrast, in Exp. 2, we wanted to determine the implications of reducing NE density in levels previously reported to impair ADG and increase fat deposition (2.29 Mcal/kg) in a synthetic line [23]. However, the low NE density in Exp. 2 was chosen to be even lower because of the high ADFI of the Duroc sire line. In this work, NE density of the diet was reduced by partly removing added fat (animal fat or palm oil, exchanged just for logistic reasons) and increasing the inclusion of high fibrous ingredients (wheat middlings), which could physically limit NE intake at high inclusions [36]. Adding fat is the most common method used to increase energy density [14,21,27,37], as its value is around 2.5–3 times that of cereal grains [35,38].

The results presented showed that in both experiments the calculated NE intake was limited when pigs were offered a low NE diet. In Exp. 1, although NE density was reduced by 7.5%, pigs could only increase ADFI by 4.5% (0.05 kg), and, therefore, the calculated NE intake was 3.2% lower in the low NE diet. Similar results were observed in the growing phase in Exp. 2. A 10.2% reduction in NE resulted in ADFI increasing by 3.5% (0.07 kg) and a 7.1% lower NE intake. In contrast, in the finishing phase, reducing NE by 9.0% resulted in a 6.9% increase in ADFI and only a 3.1% reduction in NE intake. Therefore, reducing dietary NE results in an increased ADFI [39], but it negatively affects daily NE intake [14,26]; however, the effect is less severe in heavier/older pigs [13]. Similarly to Exp. 2, De La Llata et al. [37] only reported an effect of increasing energy density in the first growing phase (25–45 and 34–60 kg BW, for gilts and barrows, respectively), but it was not significant for later phases up to 120 kg BW. Other authors did not report an effect of reducing dietary NE on daily NE intake [19,34,40]; however, Beaulieu et al. [22] reported effects when research was carried out in research facilities but not in commercial farms. It is possible, as suggested by Nitikanchana et al. [25], that there is an extra value of adding fat to a diet in addition to its high energy content.

Another disagreement in studies is whether reducing NE density affects caloric efficiency for growth, measured either as digestible, metabolizable or net energy. Quiniou and Noblet [14] showed that although NE intake was impaired when NE concentration was reduced in a wider range of NE (1.94–2.65 Mcal NE/kg), NEE was not affected. In contrast, in this study, both experiments showed an improved NEE in the low NE diets, as less calories were needed per kg gain. These results were explained based on no evidence (Exp. 1) or little effect (Exp. 2, −1.4%) of dietary NE on ADG, but a rather significant effect on NE intake. Other studies have also reported improvements in caloric efficiency when dietary NE is reduced [26,41,42]. The unexpected effect on NEE might be attributed to an additional effect of fibrous ingredients, such as wheat middlings, by limiting physical satiety [13,43] or to an underestimation or overestimation of the NE value of some ingredients [26,36]. For instance, Kil et al. [44] reported low NE values for animal fat (5.90 Mcal/kg) compared to this study (7.56 Mcal/kg). Our value was closer to those provided in different ingredient composition tables [35,38,45]. In addition, as indicated in the NRC [35], part of the discrepancies in the NE value of feed ingredients might be related to using prediction equations that were developed using complete diets.

This study did not report any relevant interaction between dietary NE and SID Lys levels used in the two factorial arrangements. Interactions could be expected in Exp. 1, in which 1.00% SID Lys limited growth performance. Thus, the results indicate that the difference in NE intake was not sufficient to limit available NE for protein deposition, or that pigs were still in the lysine-dependent phase [24]. For instance, taking the low SID Lys and high NE diet in Exp. 1 as a reference, increasing SID Lys had a greater impact on the SID Lys:NE ratio than reducing NE (0.79 vs. 0.34 g/Mcal). On the contrary, Marçal et al. [26] showed no effect of NE on ADG when diets were formulated with the same SID Lys and not based on SID Lys:NE. However, as in the present study SID Lys was formulated for the high NE diet, SID Lys intake was even higher in the low NE diets. Therefore, it was ensured that SID Lys was not limiting when NE was reduced. It is possible that if SID Lys:NE had been kept constant, reducing NE would have represented a greater negative effect on ADG because of the lower NE intake [27]. In addition, increasing SID Lys did not improve growth performance in Exp. 2, as SID Lys daily intake in the low level was greater than recommended, especially in the finishing phase [35] because of a higher ADFI than expected in the experimental design phase. Main et al. [46] suggested that around 20 g SID Lys/kg gain already maximized growth performance. In Exp. 2, this ratio was ≥19.4 g/kg, and, consequently, it did not limit growth performance.

The results in Exp. 1 provided further evidence that growing pigs fed low NE diets will have a greater ADFI if fed a common diet in the finishing phase [39]. Therefore, NE density in the growing phase could be adjusted to maximize feed intake in the finishing phase if necessary. Similarly, although it was not the aim of Exp. 1, the lower F/G in the finishing phase observed in pigs fed the low SID Lys diet between 20–40 kg BW could be related to an effect similar to compensatory growth [47]. However, it could be that if SID Lys in the common diets was not sufficiently high, only F/G would improve, but not ADG. Interestingly, in Exp. 2, the reduction in NE from 2450 to 2200–2230 kcal/kg was sufficient to influence ADG. As different NE levels were applied in each experiment, it is not possible to compare the effects in each experiment. However, in different studies, Cámara et al. [23,34] also reported different results although working in rather similar NE ranges, which could be related to the different sire lines or sex in each experiment. For instance, in one study [23], they observed a greater effect of NE on barrows ADG than boars or gilts, which we did not observe in our results.

The results on carcass composition from Exp 2. confirmed that the lower ADG observed when NE was reduced was the result of a lower fat deposition. Thus, when NE intake was limiting, the animals prioritized protein deposition above fat. Although carcass traits were only measured in the first marketed pigs, we hypothesize that a similar effect would be observed in last marketed pigs because no interaction between BW block and NE on ADG was observed for the overall period. Similarly, other studies reporting a reduction in ADG when NE density was reduced also found an effect on BFT [21,23,27]; however, others found significant effects on ADG but not on BFT [37]. Therefore, considering that a minimum HFT for pigs in Exp. 2 is required for a correct dry-curing process [4,6], feeding low NE diets might not be a good alternative in this production system.

Finally, using low NE diets reduced feed cost per kg gain when pigs did not have a huge increase in ADFI. Otherwise, the high ADFI entails that the increase in cost for the greater amount of feed required for growth is higher than the reduction in cost associated with including lower amounts of fat. For instance, in Exp. 1 (20–40 kg BW) and in Exp. 2 (32–74 kg BW), reducing dietary NE reduced feed cost per kg gain. In contrast, in Exp. 2 (74–121 kg BW), it increased feed cost per kg gain because ADFI increased by 6.9%. If the highest ADG is included by calculating the income over marginal feed costs, then the high NE density would probably result in an even better economical yield in Exp. 2 [48]. Although these economical results are only valid in the price context when the experiments were carried out, they are useful for visualizing the economic consequences of modifying dietary NE.

## 5. Conclusions

The present work provides evidence that grow-finishing pigs can partly overcome a 190–250 kcal/kg reduced dietary NE by increasing ADFI, and therefore limit the negative impact on ADG. However, no significant interaction between NE and SID Lys was reported, and the latter only showed a positive effect in pigs with a low ADFI. Reducing NE concentration was only economically feasible when feed cost was substantially lowered, and pigs did not increase ADFI in the same proportion by which NE was reduced. Furthermore, when low dietary NE density had a negative effect on ADG, this also had consequences on carcass quality by reducing fat deposition. Finally, an increased ADFI carry-over effect related to low NE diets was observed when pigs were fed a common NE diet.

## Figures and Tables

**Table 1:** Ingredient, calculated and analyzed composition (as fed basis) of the feeds used in Experiment 1.

Net Energy, kcal/kg	1.00 % SID Lys ^1^			1.20 % SID Lys	
	2360	2550		2360	2550
**Ingredient composition, %**					
Maize	37.15	35.01		36.01	33.68
Wheat	35.00	35.00		35.00	35.00
Wheat middlings	2.80	-		2.04	-
Soybean meal	19.50	21.00		20.60	21.50
Animal fat	1.00	4.50		1.00	4.50
Calcium carbonate	0.64	0.63		0.66	0.63
Dicalcium phosphate	1.24	1.27		1.25	1.27
Sodium chloride	0.42	0.42		0.42	0.42
Lysine sulphate	0.65	0.60		0.98	0.96
L-Threonine	0.18	0.17		0.30	0.29
DL-Methionine	0.15	0.15		0.26	0.27
L-Valine	0.02	-		0.13	0.13
L-Tryptophan	0.01	0.01		0.05	0.05
L-Isoleucine	-	-		0.05	0.05
Phytase ^2^	0.01	0.01		0.01	0.01
Acids mix ^3^	0.70	0.70		0.70	0.70
VIT-MIN premix ^4^	0.55	0.55		0.55	0.55
**Calculated composition ^1^**					
Dry matter, %	87.96	88.39		88.05	88.49
Crude fiber, %	3.01	2.73		2.95	2.72
Neutral detergent fiber, %	10.06	8.92		9.77	8.86
Starch, %	44.68	42.72		43.82	41.89
Crude fat, %	3.23	6.57		3.18	6.54
Crude protein, %	16.71	16.77		17.59	17.51
SID Lys, %	1.00	1.00		1.20	1.20
Net energy, kcal/kg	2360	2550		2360	2550
Total Ca	0.62	0.62		0.63	0.63
STTD P	0.38	0.38		0.39	0.38
**Analyzed composition, %**					
Crude fat	3.3	6.2		3.3	6.5
Crude protein	16.5	17.0		17.1	17.5
Lysine	1.12	1.11		1.32	1.28
Methionine + Cysteine	0.66	0.66		0.79	0.78
Threonine	0.76	0.77		0.91	0.88
Valine	0.77	0.79		0.90	0.89
Isoleucine	0.68	0.71		0.73	0.75

^1^ SID: standardized ileal digestible; STTD: standardized total tract digestible. ^2^ 6-phytase (750 FTU/kg). ^3^ Blend of formic and lactic acid with medium chain fatty acids. ^4^ Provided per each kg of feed: 6,000 IU vitamin A, 2000 IU vitamin D_3_, 20 mg vitamin E, 0.7 mg vitamin K, 1.0 mg vitamin B_1_, 4.0 vitamin B_2_, 1.2 vitamin B_6_, 0.02 vitamin B_12_, 15 mg niacin, 12 mg pantothenic acid, 120 mg choline from choline chloride, 90 mg Fe from iron sulphate, 100 mg Zn from zinc sulphate, 50 mg Mn from manganese oxide, 90 mg Cu from copper sulphate, 1.8 mg I from potassium iodide and 0.25 mg Se from sodium selenite.

**Table 2:** Ingredient, calculated and analyzed composition (as fed basis) of the feeds used in Experiment 2.

	Growing Phase (d 0–43)						Finishing Phase (d 43–85)				
SID Lysine, % ^1^	0.94			1.04			0.80			0.90	
Net Energy, kcal/kg	2200	2450		2200	2450		2230	2450		2230	2450
**Ingredient composition, %**											
Maize	30.00	30.50		30.00	30.50		25.00	40.50		25.00	40.50
Wheat	15.00	30.00		15.00	30.00		25.00	25.00		25.00	25.00
Barley	21.55	14.46		22.01	13.82		26.79	15.33		27.31	15.35
Wheat middlings	12.00	-		12.00	-		8.00	-		8.00	-
Soybean meal 47%	10.90	11.80		10.00	12.00		3.50	7.50		2.50	7.00
Sunflower meal	6.00	6.00		6.00	6.00		8.00	6.00		8.00	6.00
Animal fat	1.00	3.60		0.85	3.55		-	-		-	-
Palm oil	-	-		-	-		0.80	2.80		0.80	2.80
Calcium carbonate	1.26	1.24		1.26	1.24		1.28	1.22		1.26	1.22
Dicalcium phosphate	0.10	0.16		0.10	0.16		-	0.12		-	0.13
Sodium chloride	0.40	0.40		0.40	0.40		0.40	0.40		0.40	0.40
Lysine sulphate	0.77	0.80		1.01	0.99		-	-		-	-
Lysine HCl	-	-		-	-		0.54	0.49		0.70	0.63
L-Threonine	0.19	0.19		0.28	0.26		0.18	0.16		0.26	0.24
Liquid MHA ^2^	0.13	0.14		0.21	0.21		0.10	0.10		0.17	0.17
L-Valine	0.04	0.05		0.12	0.12		0.03	0.03		0.12	0.11
L-Tryptophan	0.02	0.03		0.04	0.04		0.02	0.02		0.05	0.04
L-Isoleucine	-	-		0.08	0.08		0.04	-		0.11	0.07
Phytase ^3^	0.02	0.02		0.02	0.02		0.02	0.02		0.02	0.02
Acids mix ^4^	0.30	0.30		0.30	0.30		-	-		-	-
VIT-MIN premix ^5^	0.32	0.32		0.32	0.32		0.32	0.32		0.32	0.32
**Calculated composition**											
Dry matter, %	88.1	88.5		88.1	88.3		88.6	88.5		88.7	88.5
Crude fiber, %	5.49	4.44		5.47	3.63		5.57	4.30		5.55	4.28
Neutral detergent fiber, %	16.1	12.0		16.1	10.7		16.1	11.8		16.1	11.8
Starch, %	41.1	44.2		41.3	45.2		45.8	48.0		46.1	48.0
Crude fat, %	3.20	5.54		3.05	4.79		2.90	4.94		2.90	4.93
Crude protein, %	15.6	15.1		15.7	15.7		13.4	13.3		13.4	13.4
SID Lys, %	0.94	0.94		1.04	1.04		0.80	0.80		0.90	0.90
Net energy, kcal/kg	2200	2450		2200	2450		2230	2450		2230	2450
Total Ca	0.60	0.60		0.60	0.60		0.57	0.57		0.56	0.57
STTD P ^6^	0.31	0.30		0.31	0.30		0.29	0.29		0.29	0.29
**Analyzed composition, %**											
Crude fat	3.5	5.5		3.4	4.9		3.3	5.2		3.2	5.3
Crude protein	15.9	15.8		16.0	16.3		13.6	13.4		13.7	13.8
Lysine	1.04	1.07		1.19	1.18		0.91	0.87		0.99	0.97
Methionine + Cysteine ^7^	0.61	0.64		0.70	0.71		0.56	0.54		0.61	0.60
Threonine	0.67	0.70		0.77	0.77		0.61	0.59		0.65	0.63
Valine	0.72	0.75		0.81	0.83		0.64	0.63		0.71	0.70
Isoleucine	0.55	0.60		0.63	0.68		0.50	0.49		0.55	0.54
**Feed cost, €/t**	220.2	232.8		231.1	249.0		204.0	212.6		219.8	228.6

^1^ SID: standardized ileal digestible. ^2^ Methionine hydroxy analogue. ^3^ 6-phytase (750 FTU/kg). ^4^ Blend of formic and lactic acid with medium chain fatty acids. ^5^ Provided per each kg of feed in the growing phase: 4500 IU vitamin A, 2000 IU vitamin D_3_, 15 mg vitamin E, 0.7 mg vitamin K, 1.0 mg vitamin B_1_, 4.0 vitamin B_2_, 1.2 vitamin B_6_, 0.020 vitamin B_12_, 15 mg niacin, 12 mg pantothenic acid, 107 mg of choline from choline chloride, 90 mg Fe from iron sulphate, 100 mg Zn from zinc sulphate, 50 mg Mn from manganese oxide, 20 mg Cu from copper sulphate, 1.8 mg I from potassium iodide and 0.25 mg Se from sodium selenite. Provided per each kg of feed in the finishing phase: 2000 IU vitamin A, 1500 IU vitamin D_3_, 7 mg vitamin E, 0.6 mg vitamin K, 0.8 mg vitamin B_1_, 3.2 vitamin B_2_, 1.0 vitamin B_6_, 0.016 vitamin B_12_, 12 mg niacin, 7 mg pantothenic acid, 107 mg choline from choline chloride, 72 mg Fe from iron sulphate, 80 mg Zn from zinc sulphate, 40 mg Mn from manganese oxide, 16 mg Cu from copper sulphate, 1.8 mg I from potassium iodide and 0.25 mg Se from sodium selenite. ^6^ STTD: standardized total tract digestible. ^7^ Sum of Met and Cys from vegetal sources and synthetic Met from methionine hydroxy analogue.

**Table 3:** Effects of increasing dietary standardized ileal digestible lysine (SID Lys) and reducing net energy (NE) concentration on growth performance of Pietrain grow-finishing pigs when fed the experimental treatments or a common diet (Experiment 1).

	NE (kcal/kg)	2360			2550		SEM ^2^	*p*-Value ^1^		
Item ^3^	SID Lys (%)	1.00	1.20		1.00	1.20		Lys	NE	Lys × NE
**Body weight, kg**										
d 0		19.8	19.9		19.8	19.9	0.15	0.847	0.962	0.966
d 14 ^4^		28.6	29.4		28.6	29.2	0.64	0.003	0.556	0.741
d 26 ^5^		37.6	38.7		37.4	38.5	0.50	<0.001	0.331	0.968
d 68 ^5^		68.4	69.6		68.1	69.0	0.44	0.023	0.328	0.754
d 116 ^5^		109.7	110.3		107.9	109.4	0.70	0.103	0.025	0.450
***Phase 1*, d 0–14**										
ADG, kg		0.630	0.680		0.625	0.666	0.0386	<0.001	0.293	0.621
ADFI, kg		1.000	1.010		0.964	0.961	0.0430	0.857	<0.001	0.617
SID Lys intake, g/d		10.0	12.1		9.6	11.5	0.47	<0.001	<0.001	0.405
NE intake, Mcal/d		2.37	2.38		2.46	2.45	0.105	0.870	0.003	0.615
Feed/gain		1.59	1.49		1.54	1.44	0.028	<0.001	0.001	0.730
NEE, Mcal/kg		3.76	3.51		3.93	3.68	0.068	<0.001	<0.001	0.967
LysE, g/kg		15.9	17.8		15.4	17.3	0.30	<0.001	<0.001	0.983
Feed cost/gain, EUR/kg		0.403	0.394		0.414	0.404	0.0074	0.006	0.004	0.979
***Phase 2*, d 14–26**										
ADG, kg ^5^		0.740	0.775		0.727	0.773	0.0122	<0.001	0.432	0.545
ADFI, kg		1.31	1.31		1.25	1.26	0.013	0.837	<0.001	0.898
SID Lys intake, g/d		13.1	15.8		12.5	15.1	0.14	<0.001	<0.001	0.775
NE intake, Mcal/d		3.10	3.10		3.20	3.21	0.031	0.831	0.002	0.890
Feed/gain ^4^		1.77	1.70		1.72	1.63	0.024	<0.001	<0.001	0.442
NEE, Mcal/kg ^4^		4.19	4.00		4.40	4.15	0.060	<0.001	<0.001	0.291
LysE, g/kg ^4,6^		17.7	20.3		17.2	19.5	0.26	<0.001	<0.001	0.220
Feed cost/gain, EUR/kg ^4^		0.449	0.449		0.462	0.455	0.0065	0.266	0.003	0.239
***Phase 1* & *2*, d 0–26**										
ADG, kg		0.681	0.724		0.672	0.715	0.0171	<0.001	0.220	0.993
ADFI, kg^4^		1.15	1.15		1.10	1.10	0.025	0.821	<0.001	0.826
SID Lys intake, g/d ^4^		11.5	13.8		11.0	13.2	0.27	<0.001	<0.001	0.505
NE intake, Mcal/d ^4^		2.70	2.71		2.80	2.80	0.060	0.825	<0.001	0.831
Feed/gain		1.68	1.59		1.63	1.53	0.008	<0.001	<0.001	0.837
NEE Mcal/kg		3.97	3.74		4.16	3.91	0.019	<0.001	<0.001	0.474
LysE, g/kg		16.8	19.0		16.3	18.4	0.09	<0.001	<0.001	0.397
Feed cost/gain, EUR/kg ^7^		0.425	0.420		0.437	0.429	0.0020	0.001	<0.001	0.385
**Common diet, d 26–116**										
ADG, kg ^6^		0.801	0.794		0.785	0.787	0.0055	0.695	0.037	0.371
ADFI, kg		1.79	1.79		1.74	1.75	0.013	0.491	0.001	0.843
Feed/gain		2.23	2.26		2.22	2.23	0.010	0.059	0.024	0.274
**Overall d 0–116**										
ADG, kg ^5^		0.788	0.790		0.772	0.784	0.0045	0.106	0.020	0.265
ADFI, kg		1.64	1.65		1.60	1.61	0.014	0.491	<0.001	0.892
Feed/gain ^5^		2.09	2.09		2.07	2.05	0.019	0.162	<0.001	0.100
Feed cost/gain, EUR/kg ^7^		0.473	0.477		0.474	0.474	0.0016	0.310	0.454	0.145

Least square means. ^1^ Statistical model included the effects of SID Lys, NE, initial BW block, sex, and all double interactions between these factors. Lys = SID Lys. ^2^ SEM: standard error of the mean. ^3^ ADG: average daily gain; ADFI: average daily feed intake; NEE: net energy efficiency per kg BW gain; LysE: SID Lys intake per kg gain. ^4^ BW block × sex interaction (*p* < 0.05). ^5^ NE × BW block interaction (*p* < 0.05). ^6^ SID Lys × BW block interaction (*p* = 0.036). ^7^ SID Lys × sex interaction (*p* < 0.05).

**Table 4:** Effects of increasing dietary standardized ileal digestible lysine (Lys) and reducing net energy (NE) concentration on growth performance of Duroc grow-finishing pigs (Experiment 2).

	Day 0–43	2200 kcal NE/kg			2450 kcal NE/kg		SEM ^2^	*p*-Value ^1^		
	SID Lys, %	0.94	1.04		0.94	1.04				
	Day 43–85	2230 kcal NE/kg			2450 kcal NE/kg			Lys	NE	Lys × NE
Item ^3^	SID Lys, %	0.80	0.90		0.80	0.90				
**Body weight, kg**										
d 0		32.5	32.4		32.4	32.5	0.13	0.890	0.939	0.601
d 23 ^4^		50.9	51.4		51.5	51.3	0.72	0.644	0.513	0.269
d 43		74.0	74.2		75.1	74.5	0.68	0.717	0.157	0.476
d 72		107	108		109	109	0.88	0.903	0.039	0.943
d 85 ^5^		120	121		122	122	1.16	0.714	0.077	0.867
***Growing phase*, d 0–43**										
ADG, kg ^6,7^		0.956	0.969		0.985	0.972	0.0135	0.992	0.105	0.213
ADFI, kg ^5^		2.07	2.11		2.03	2.01	0.028	0.469	<0.001	0.102
SID Lys intake, g/d ^5^		19.4	22.0		19.1	20.9	0.28	<0.001	<0.001	0.071
NE intake, Mcal/d		4.54	4.65		4.97	4.92	0.065	0.526	<0.001	0.111
Feed/gain ^8^		2.16	2.18		2.06	2.07	0.011	0.295	<0.001	0.605
NE/gain, Mcal/kg		4.75	4.79		5.05	5.06	0.026	0.307	<0.001	0.645
SID Lys/gain, g/kg ^5,8^		20.3	22.6		19.4	21.5	0.11	<0.001	<0.001	0.322
Feed cost/gain, EUR/kg ^5,8^		0.475	0.503		0.479	0.514	0.0026	<0.001	0.004	0.166
***Finishing phase*, d 43–85**										
ADG, kg		1.099	1.099		1.106	1.119	0.0136	0.570	0.220	0.555
ADFI, kg		3.25	3.28		3.05	3.08	0.034	0.296	<0.001	0.889
SID Lys intake, g/d ^5^		26.0	29.5		24.4	27.7	0.30	<0.001	<0.001	0.811
NE intake, Mcal/d		7.25	7.31		7.46	7.55	0.078	0.287	0.002	0.849
Feed/gain		2.96	2.98		2.75	2.76	0.016	0.362	<0.001	0.482
NE/gain, Mcal/kg		6.60	6.65		6.74	6.75	0.037	0.380	0.001	0.510
SID Lys/gain, g/kg		23.7	26.9		22.0	24.8	0.14	<0.001	<0.001	0.141
Feed cost/gain, EUR/kg		0.604	0.656		0.585	0.630	0.0035	<0.001	<0.001	0.276
***Overall*, d 0–85**										
ADG, kg ^5,7,9^		1.027	1.033		1.045	1.044	0.0116	0.637	0.027	0.610
ADFI, kg ^5^		2.65	2.69		2.53	2.54	0.028	0.250	<0.001	0.483
SID Lys intake, g/d ^5^		22.7	25.7		21.7	24.3	0.26	<0.001	<0.001	0.249
NE intake, Mcal/d ^5^		5.88	5.96		6.20	6.20	0.064	0.260	<0.001	0.516
Feed/gain		2.58	2.60		2.42	2.43	0.010	0.184	<0.001	0.668
NE/gain, Mcal/kg		5.72	5.77		5.93	5.95	0.024	0.192	<0.001	0.721
SID Lys/gain, g/kg		22.1	24.8		20.7	23.2	0.09	<0.001	<0.001	0.144
Feed cost/gain, EUR/kg		0.541	0.579		0.533	0.574	0.0025	<0.001	0.004	0.675

Least square means. ^1^ Statistical model included the effects of Lys, NE, initial BW block, sex, all double interactions between these factors and the triple interaction between Lys, BW BLOCK and sex. ^2^ SEM: standard error of the mean. ^3^ ADG: average daily gain; ADFI: average daily feed intake; SID Lys: standardized ileal digestible lysine. ^4^ BW BLOCK × sex interaction (*p* < 0.05). ^5^ Lys × sex interaction (*p* < 0.05). ^6^ NE × BW BLOCK interaction (*p* < 0.05). ^7^ Lys × BW BLOCK × sex interaction (*p* < 0.05). ^8^ NE × sex interaction (*p* < 0.05). ^9^ Lys x BW BLOCK interaction (*p* < 0.05).
